# Differences in the phenotypes and transcriptomic signatures of chimeric antigen receptor T lymphocytes manufactured *via* electroporation or lentiviral transfection

**DOI:** 10.3389/fimmu.2023.1068625

**Published:** 2023-05-09

**Authors:** Anna Niu, Jintao Zou, Xuan Hu, Zhang Zhang, Lingyu Su, Jing Wang, Xing Lu, Wei Zhang, Wei Chen, Xiaopeng Zhang

**Affiliations:** ^1^Beijing Institute of Biotechnology, Beijing, China; ^2^Nanhu Laboratory, Jiaxing, Zhejiang, China

**Keywords:** chimeric antigen receptor T lymphocyte, piggyBac transposon, lentiviral transfection, phenotype, transcriptomic signature

## Abstract

Chimeric antigen receptor (CAR)-T cell therapy is an innovative treatment for CD19-expressing lymphomas. CAR-T cells are primarily manufactured via lentivirus transfection or transposon electroporation. While anti-tumor efficacy comparisons between the two methods have been conducted, there is a current dearth of studies investigating the phenotypes and transcriptome alterations induced in T cells by the two distinct manufacturing methods. Here, we established CAR-T signatures using fluorescent imaging, flow cytometry, and RNA-sequencing. A small fraction of CAR-T cells that were produced using the PiggyBac transposon (PB CAR-T cells) exhibited much higher expression of CAR than those produced using a lentivirus (Lenti CAR-T cells). PB and Lenti CAR-T cells contained more cytotoxic T cell subsets than control T cells, and Lenti CAR-T cells presented a more pronounced memory phenotype. RNA-sequencing further revealed vast disparities between the two CAR-T cell groups, with PB CAR-T cells exhibiting greater upregulation of cytokines, chemokines, and their receptors. Intriguingly, PB CAR-T cells singularly expressed IL-9 and fewer cytokine release syndrome-associated cytokines when activated by target cells. In addition, PB CAR-T cells exerted faster *in vitro* cytotoxicity against CD19-expressing K562 cells but similar *in vivo* anti-tumor efficacy with Lenti CAR-T. Taken together, these data provide insights into the phenotypic alterations induced by lentiviral transfection or transposon electroporation and will attract more attention to the clinical influence of different manufacturing procedures.

## 1 Introduction

Chimeric antigen receptor (CAR)-T cell therapy has been revolutionized in hematologic malignancies with the recent development and emergence of adoptive cell therapy. CARs consist of two major functional components: extracellular recognition and intracellular signal transduction molecules. CARs comprise a single chain variable fragment (scFv), transmembrane region, co-stimulation signaling domain, CD28 (1) or 4-1BB (2) domain, and CD3ζ domain, which elicit profound and durable anti-B cell leukemia responses (3). CAR-T cell therapy targeting CD19 was first approved by the US Food and Drug Administration in 2017 (4). The overall response rate of patients with B cell acute lymphoblastic leukemia is 73%–83% (5–7) with an annual cost of up to $1,615,000 (8). However, side effects, cytokine release syndrome (9), and neurotoxicity (10) caused by CAR-T cells are concerning barriers, and some patients achieve only about 50% remission after receiving CAR-T cell therapy (11, 12). To achieve an optimized risk/benefit ratio in patients receiving CAR-T cell therapy, all factors affecting antigen binding, exhaustion, duration, and signaling activation should be considered during the design and manufacturing process, as even slight alterations in the CAR design will alter the function and side effects of CAR-T cell therapy (13).

The CAR gene transfer method may represent a critical factor that affects the phenotype of CAR-T cells. The predominant manufacturing procedures have been confirmed to be safe and effective and primarily involve lentiviral/retroviral transfection or transposon electroporation (14, 15). Although replication-competent lentivirus/retrovirus have been shown to cause oncogenesis of gene-modified cells, data for 375 manufactured T cell products with self-inactivated lentivirus/retrovirus exhibited low safety risk for HIV and oncology patients (16). The lentiviral/retroviral transfection system packages RNA encoding transgenes and essential viral genome components, such as the Rev responsive element (RRE), 5′ long terminal repeat (LTR), 3′ LTR, and Psi elements (17). Following infection of target cells, the RNA is reverse transcribed into DNA and subsequently integrated into the cell genome (18). Transposon systems generally comprise two vectors encoding the enzyme and transgenes. In comparison, when electroporated into cells and expressed, transposase excises transposons from the plasmid and integrates them into the target genome. In particular, the PiggyBac transposon system can efficiently transpose between vectors and chromosomes via a “no footprint cut-and-paste” mechanism. Recently, electroporation of CAR plasmids into T cells was introduced, and their safety and efficacy were assessed (19). We previously reported an optimized electroporation method for constructing functional CAR-T cells (20). In phase I clinical trials, the transposon system achieved a 2200–2500 fold expansion of CAR-T cells with 84% positivity after co-culturing with feeder cells (19). Moreover, the use of minicircle vectors in this system was less likely to cause genomic damage during mutagenesis (21).

Lentivirus and transposon systems expose T cells to considerably different stimuli. More specifically, the integrated fragments differ due to the essential viral genome compounds required by the lentivirus for reverse transcription and nuclear translocation, whereas reverse transcription is not required in the transposon system. Additionally, the mode of entry into the cells (viral infection versus electroporation) differs between the two methods.

Functional comparisons of CAR-T cells produced using the two manufacturing processes have been conducted in mouse xenograft models by different groups (21, 22) and have shown similar anti-tumor efficacy; however, there is a lack of comprehensive data on the perturbation of intracellular signaling networks and transcriptomes of T cells subjected to the distinct manufacturing methods. Hence, in the current study, we sought to explore phenotypic differences between CAR-T cells produced via lentiviral transfection (Lenti CAR-T cells) or PiggyBac transposon electroporation (PB CAR-T cells) using transcriptome analysis and flow cytometry. We then determined the potential effects of these differences on the anti-tumor efficacy of CAR-T cells.

## 2 Materials and methods

### 2.1 Primary cells and cell lines

Peripheral blood mononuclear cells (PBMCs) were isolated by density gradient centrifugation using Ficoll Paque Plus (Cytiva, USA) from whole blood samples obtained from healthy donors. The PBMCs were cultured in Xvivo 15 medium (Lonza, Belgium), and cryopreserved in fetal bovine serum (FBS) containing 10% dimethyl sulfoxide. CD19-expressing luciferase-tagged K562 cells (Shanghai Genechem Co., Ltd.) were cultured in Iscove’s Modified Dulbecco’s Medium (Lonza, Belgium) supplemented with 10% FBS and used as target cells for the assessment of CAR-T cell cytotoxicity. Raji cells were cultured in RPMI 1640 medium supplemented with 10% FBS and used for the assessment of CAR-T cell efficacy *in vivo*.

### 2.2 Construction of CD19-targeting CAR transposon and lentiviral vectors

Constructs containing CD19-targeting CAR molecules—including a CD8a signal peptide, clone FMC63 CD19-targeting scFv, CD8a transmembrane domain, 4-1BB domain, and CD3ζ domain—and TagGFP2 separated by a P2A sequence, were produced. CAR expression was controlled by the EF-1α promoter. The consensus EF-1α promoter and CD19-targeting CAR open reading frame (ORF) were cloned into the pLenti lentiviral gene expression vector (Origene, USA) and PiggyBac dual promoter vector (System Biosciences, USA).

### 2.3 Electroporation of the CD19-targeting CAR transposon

Electroporation was performed as previously described (20), with slight modifications. In brief, PBMCs were stimulated with anti-CD3/CD28-coated beads (Thermo Fisher Scientific, USA) at a bead-to-cell ratio of 1:1 for 3 days. Then cells were counted and washed twice to remove the beads. Next, 1 × 10^6^ primary T cells were resuspended with 2.1 μg plasmids in 20 μL electroporation buffer containing approximately 0.7 μg of the Super PiggyBac transposase vector and 1.4 μg of the CD19-targeting CAR transposon vector. The resulting mixture was immediately transferred to 20-μL electroporation tubes and subjected to electroporation condition (voltage = 500 V, time = 20 ms) within an electroporator (Celetrix CTX-1500A LE, USA) and then gently transferred into pre-warmed Xvivo 15 medium without antibiotics.

### 2.4 Manufacturing of CD19-targeting CAR-T cells *via* lentiviral transfection

Lentivirus generation was performed as described previously (22), with slight modifications. Second-generation lentiviral vectors were also produced. The pLenti CD19-targeting CAR vector was co-transfected into 293T cells with the packaging vector and the spike glycoprotein of the vesicular stomatitis virus (VSV-G)-expressing vector. Lentivirus was concentrated from the medium supernatant with a lentivirus concentrator kit (Oligobio, China), detected via flow cytometry, resuspended in phosphate buffer saline without Mg^2+^ and Ca^2+^, and frozen at -80°C. PBMCs were cultured for 24 hours before activation, suspended at a concentration of 1 × 10^6^ cells/mL, and incubated with anti-CD3/CD28-coated beads (Thermo Fisher Scientific, USA) at a bead-to-cell ratio of 1:1. After activation, T cells were infected with lentivirus at a multiplicity of infection of 3.0. The media was refreshed 24 hours post-transfection.

### 2.5 Flow cytometry analysis

1 × 10^6^ CAR-T cells were stained with protein L labeled with iFluor647 (GenScript, USA). iFluor647 was detected using GUAVA easyCyte HT after separate detection of dead cells using acridine orange/propidium iodide staining. Analysis of CAR-T cells was conducted using the method described by Blom et al. (23). In brief, manufactured T cells were stained with live/dead dye from the Zombie Aqua Fixable Viability Kit (BioLegend) and primary antibodies, according to manufacturers’ protocols, including Alexa Fluor 700 conjugated anti-human CD3 antibody (Clone UCHT1), PE-Cy7 conjugated anti-human CD4 antibody (Clone RPA-T4), PerCP-Cy5.5 conjugated anti-human CD8 antibody (Clone SK1), APC conjugated anti-human CD69 antibody (Clone FN50), Brilliant Violet 605 anti-human CD62L antibody (Clone DREG-56), PE/Dazzle 594 anti-human CD45RO antibody (Clone UCHL1), Brilliant Violet 421 anti-human PD-1 antibody (Clone EH12.2H7), APC-conjugated anti-human CD107a (Clone H4A3), and PE-conjugated anti-human Granzyme B (Clone QA18A28). Gates for CD62L, CD45RO, PD-1, and Granzyme B fluorescence were further validated using fluorescence minus one control. Peripheral blood collected from mice at 3 weeks post-infusion with T/CAR-T cells were stained with PE-Cy7 conjugated anti-human CD3 antibody (Clone UCHT1), Pacific Blue conjugated anti-human CD45 (Clone HI30), and FITC conjugated anti-human CD19 antibody (Clone HIB19). All antibodies used in this study and their corresponding isotypes were purchased from BioLegend company. The positives of each marker were gated against an isotype control. The data were analyzed using FlowJo V10.8.1.

### 2.6 RNA extraction, library construction, sequencing, and validation

The PBMCs samples were collected from healthy donors and samples from each donor were divided into three groups: control T(untreated), Lenti CAR-T, and PB CAR-T. After manufacturing, CAR-T cells were isolated with the flow cytometer Sony MA900. The samples were frozen in liquid nitrogen until RNA extraction. Total RNA was extracted from CAR-T cells using RNAiso Plus (Takara, Japan). RNA quality was evaluated by Nanodrop spectrophotometer (Thermo Fisher Scientific, USA). The NEBNext Ultra RNA Library Prep Kit for Illumina reagent was used to prepare the RNA library. The reference genome files were downloaded from genome websites (https://www.ncbi.nlm.nih.gov/genome/51?genome_assembly_id=1820449). The filtered reads were mapped to the reference genome using the HISAT2 version 2.0.5. MicroRNAs were isolated using a miRNeasy Micro Kit (QIAGEN, Germany) according to the manufacturer’s instructions. Quality was assessed with an Agilent 4200 TapeStation, and quantity was determined using a Qubit 2.0 Fluorometer (Thermo Fisher Scientific, USA). The samples were sent to Shanghai Personal Biotechnology Co,. Ltd. for mRNA library construction and sequencing on an Illumina HiSeq platform (Illumina).

To perform RT-qPCR validation, total RNA sample (1 μg) was used for synthesis of cDNA and PCR amplification using the HiScript II One Step RT-PCR Kit (Vazyme, China) as manufacturers’ protocols described. Primers for RT-qPCR analysis were synthesized by Sangon Biotech Co., Ltd. (Shanghai, China). RT-qPCR was carried out with the Bio-Rad CFX384 Touch real-time PCR detection system. The relative expression levels of the selected genes were analyzed using the comparative CT method (2^−ΔΔCT^). Each RT-qPCR analysis was repeated at least 4 times and *β-actin* was used as the reference control.

### 2.7 Principle component analysis and pathway analysis

Comparison was made between the Read Count values for each gene as the original expression of that gene. The expression was then standardized with fragments per kilobase of exon per million mapped fragments (FPKM). Differences in gene expression were analyzed with DESeq software (version 1.39.0) under the following screening conditions: log_2_ fold change > 1, *P*-value < 0.05. Principal component analysis (PCA) was then performed as described previously (24). Kyoto Encyclopedia of Genes and Genomes (KEGG) analysis was performed using differentially expressed genes with an adjusted *P*-value of ≤ 0.01. The number of differentially enriched genes in KEGG pathway was calculated. Gene Set Enrichment Analysis (GSEA, https://www.gsea-msigdb.org) was performed to investigate distinct genesets between PB and Lenti CAR-T groups.

### 2.8 Enzyme-linked immunosorbent assay

After CAR-T cells manufacturing or coculturing with target cells, cytokine analysis was performed by using ELISA, according to the manufacturers’ protocols. The concentration of IFN-γ, TNF-α, GM-CSF, CXCL10, IL-6, IL-10, IL-12, and CCL2 was measured by an Ella automated immunoassay kit (Bio-Techne, USA). Concentrations of IL-9 and Granzyme B were determined using enzyme-linked immunosorbent assay kits (Dakewei, China or Neobioscience, China).

### 2.9 *In vitro* anti-tumor efficacy

Luciferase-expressing K562 CD19 cells were co-cultured with CAR-T cells at desired effector-to-target (E:T) ratios for either 4 or 24 hours. Following co-culture, luciferin substrate was introduced to the system at a final concentration of 0.3 mg/mL. Relative luminescence units (RLUs) were measured using a SpectraMax M5 plate reader (Molecular Devices, CA). The lysis rates of tumor cells were calculated by the following formula: %tumor cell lysis =100% × (1 – RLU (experimental -background)/(target cell max-background).

The cytotoxicity of CAR-T cells was further evaluated using the xCELLigence Real-Time Cellular Analysis DP platform (Agilent Technologies, CA). This system measures a dimensionless parameter called cell index (CI) to determine the viability of the cell and tumor lysis rate. Briefly, K562-CD19-luc cells were plated at a concentration of 2.5 × 10^4^ cells per well in 200 µL of cell culture medium in 16-well E-plates. After seeding, the cells were cultured on the xCELLigence instrument in a humidified incubator at 37°C with 5% CO_2_ for 24 hours. Subsequently, T/CAR-T cells were seeded onto the E-plates at an E:T ratio of 2.5:1. Continuous impedance measurements were then monitored every 5 minutes for up to 72 hours. Four replicates were set for each group. The cell index was normalized to the time point of addition of the T/CAR-T cells. The lysis rates of tumor cells were calculated by the following formula: [1 − Normalized CI_treatment_/Normalized CI_target only_] × 100%.

### 2.10 *In vivo* anti-tumor efficacy

Six-week-old NSG (NOD-*Prkdc^scid^Il2rg^em1^*/Smoc) mice were intravenously injected with 1×10^6^ Raji cells *via* the tail vein. Two weeks after tumor cell inoculation, the mice received intravenous treatment with 1.5×10^6^ CAR-T cells. Serum samples were collected at 4 hours and 24 hours post-infusion of CAR-T cells. Peripheral blood samples were obtained three weeks post-infusion and analyzed for the presence of CD19^+^ Raji cells by flow cytometry. Animals used in this study were reviewed and approved by the Animal Care and Use Committee of the Beijing Institute of Biotechnology.

### 2.11 Statistical analysis

Data were presented as the mean ± standard deviation (SD) in triplicate. Flow cytometry data were analyzed using BD FlowJo software (version 10.8.1). To determine *P*-values, one-way ANOVA with Tukey’s multiple comparison test or student’s *t*-test was applied. GraphPad Prism 9.0 was used to perform statistical analyses.

## 3 Results

### 3.1 Production of CAR-T cells by electroporation and lentiviral transfection

**Figure 1:**
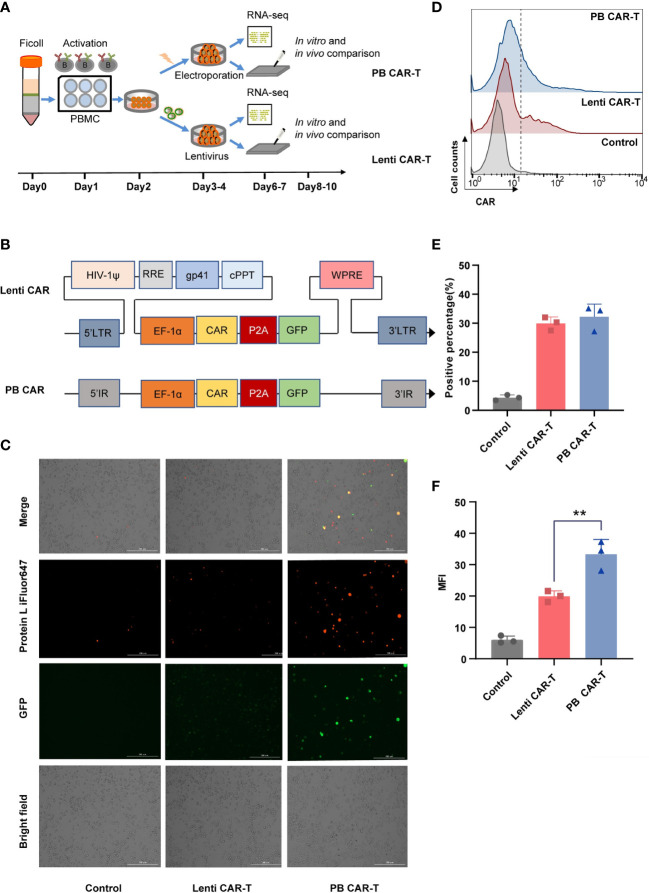
Production of CAR-T cells *via* PiggyBac transposon electroporation or lentiviral transfection. **(A)** CAR-T cell manufacturing schedule. **(B)** CAR insertion fragments used in the study. The scFv of CAR is derived from mAb clone FMC63 that binds human CD19 and was generated by fusing the VL and VH regions via a 3× G4S linker peptide. The scFv was attached to modified human CD8a hinge and CD8a transmembrane regions that were fused to the 4-1BB (cytoplasmic) and CD3ζ (cytoplasmic) domains. **(C)** Representative fluorescent images of CAR-positive T cells. Transfection efficiency after 48 hours was demonstrated by fluorescence microscopy images with Bio-Tek Cytation 5. **(D–F)** Flow cytometry detection of CAR expression on the surface of transduced CAR-T cells on day 6. Lenti CAR-T cells and PB CAR-T were generated by Lentivirus transfection and plasmid electroporation, respectively. **(D)** iFluor 647 Protein L that binds to the variable light chains of scFv can be used for the detection of CAR expression in 1 × 10^6^ cells at a 1:100 dilution ratio. Untransduced T cells were used as control groups. Representative flow cytometry plots of CAR-positive T cells. **(E)** Statistical analysis of CAR-positive percentages (*n* = 3). **(F)** Statistical analysis of mean fluorescent intensity (MFI) values represents the mean fluorescence intensity of iFluor 647 protein L (*n* = 3). One-way ANOVA with Tukey’s multiple comparison test. **P < 0.01.

T cells isolated from healthy donors were used to manufacture Lenti CAR-T cells and PB CAR-T cells. To minimize technical bias, both manufacturing processes were carried out following the same schedule (**Figure 1A**). The two vector systems share a consensus ORF and promoter (**Figure 1B**). Higher green and red fluorescent intensities of PB CAR T cells were shown in **Figure 1C**, compared with Lenti CAR-T cells. Moreover, the percentage of CAR-positive T cells was adjusted to approximately 30% for both groups on day 6 (**Figures 1D, E**). The mean fluorescence intensity (MFI) values of PB CAR-T cells were significantly higher than those of Lenti CAR-T cells (**Figure 1F**), suggesting relatively weak expression of CAR molecules following lentiviral transfection. The reduced CAR expression in Lenti CAR-T cells may be attributed to excessive viral elements integrated into the genome of the target cells.

### 3.2 Analysis of CAR-T cell cytotoxicity, activation, and exhaustion

Flow cytometry was performed to characterize the two CAR-T cell groups. The analysis pipeline was shown in **Supplementary Figure 1**. The CD4^+^ and CD8^+^ gates divided the T cells into four subsets: CD4^+^, CD8^+^, CD4^+^ CD8^+^ (double-positive, DP), and CD4^-^CD8^-^ (double-negative, DN). These subsets were further gated using CD69, granzyme B, and programmed cell death-1 (PD-1) as markers of T cell activation, cytotoxicity, and exhaustion, respectively (25). Given that the DP T cell subset only accounted for ~6% of the total T cells, markers of DP T cells were not analyzed.

**Figure 2:**
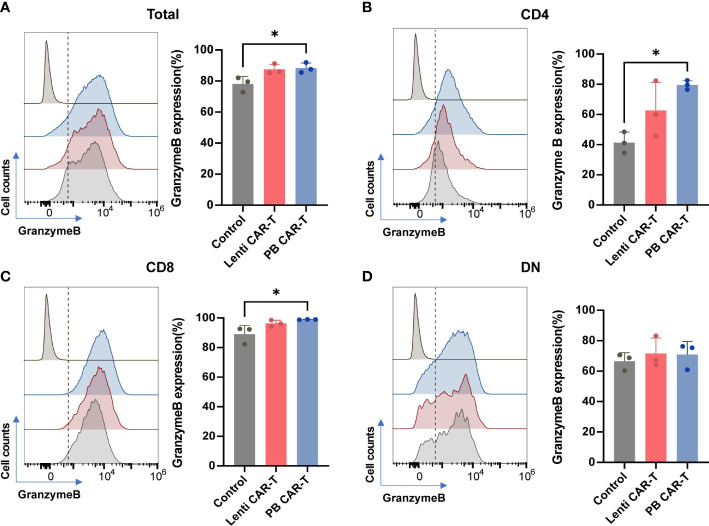
Flow cytometry analysis of granzyme B expression of total T cells and T cell subsets. CAR-T cells at day 3 post-transfection were analyzed using flow cytometry. Representative histogram images and statistical analysis of granzyme B expression in total T cells **(A)** and CD4^+^ **(B)**, CD8^+^ **(C)**, and double negative (DN) **(D)** T cell subsets (*n* = 3). Isotype controls were presented in the upper first lane. One-way ANOVA with Tukey’s multiple comparison test. *P < 0.05.

Granzyme B, secreted by cytotoxic T lymphocytes, triggers target cell DNA cleavage and apoptosis by binding to its receptor and being released by perforin (26). The proportion of granzyme B^+^ cells was consistently high, ranging from 80% to 90% across all groups (**Figures 2A–D**), as evidenced by CD107a staining (**Supplementary Figure 2A, B**). Following CAR transfection, the proportion of granzyme B^+^ cells in the PB groups slightly increased. (**Figures 2A–C**), with the CAR-T cell groups exhibiting the highest proportion of granzyme B^+^ cells in the CD8^+^ subset (**Figures 2C**). No significant difference was observed in CD69 expression between PB CAR-T cells and Lenti CAR-T cells (**Supplementary Figures 2C–2F**). Expression of PD-1, which is a member of the CD28 superfamily that negatively regulates T cells upon activation by PD-L1 or PD-L2 (27), was similar in T cells before and after CAR insertion (**Supplementary Figure 3**), indicating that neither manufacturing process induced T cell exhaustion.

### 3.3 Fewer PB CAR-T cells exhibit a memory phenotype than Control T cells

**Figure 3:**
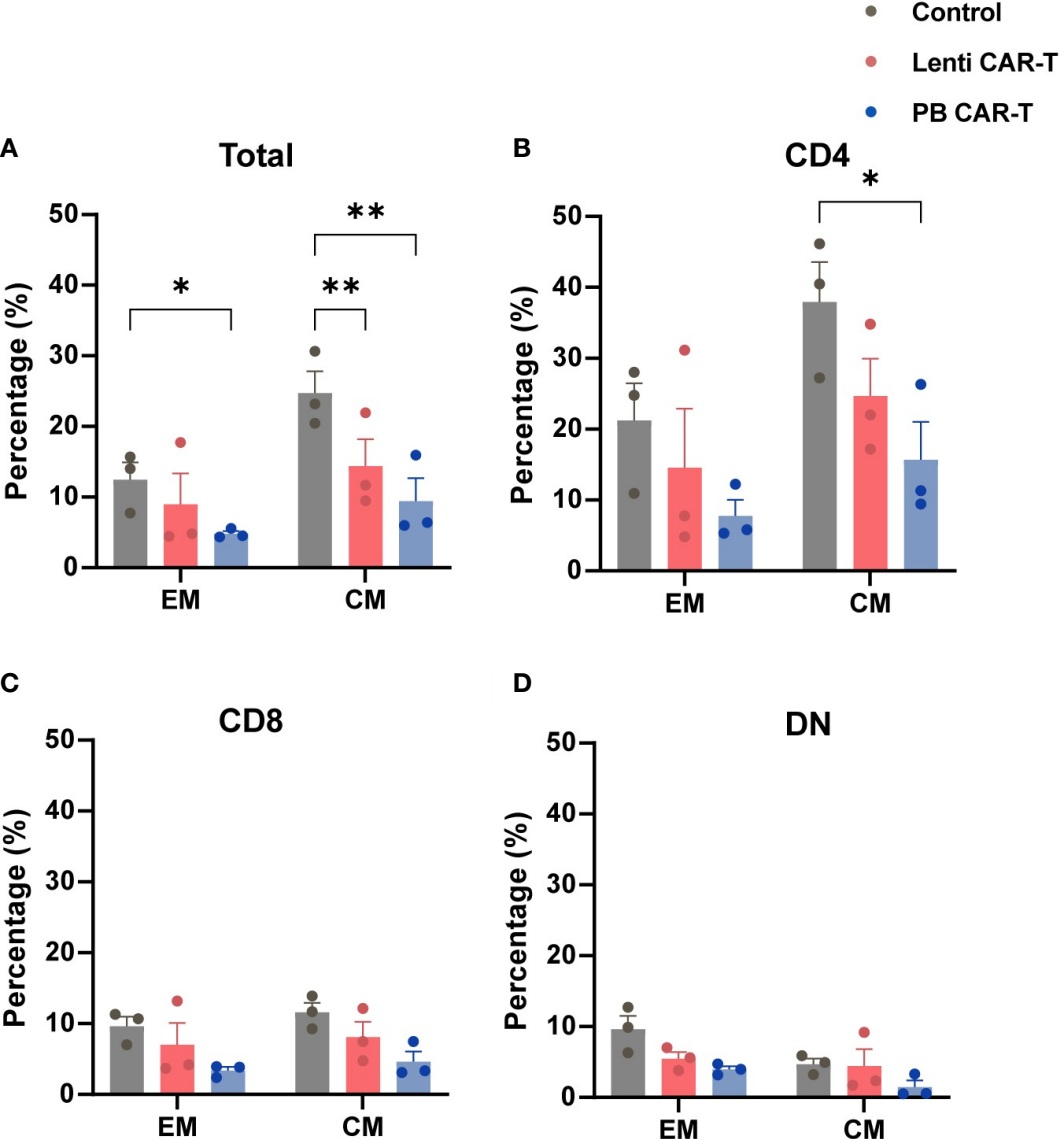
Memory phenotypes of total T cells and T cell subsets. CAR-T cells at day 3 post-transfection were analyzed using flow cytometry. T cells were stained with anti-CD45RO and anti-CD62L antibodies. Statistical analysis of CD45RO and CD62L expression in total T cells **(A)**, CD4^+^ T cells **(B)**, CD8^+^ T cells **(C)**, and DN T cells **(D)**. CD45RO^+^CD62L^+^ and CD45RO^+^CD62L^-^ represent central memory (CM) and effector memory (EM) phenotypes, respectively (*n* = 3). One-way ANOVA with Tukey’s multiple comparison test. *P < 0.05, **P < 0.01.

CD62L and CD45RO are major plasma membrane markers that distinguish central memory (CM; CD45RO^+^CD62L^+^) T cells from effector memory (EM; CD45RO^+^CD62L^-^) T cells (23). T_EM_ function as rapid effectors by migrating to inflamed sites through surface chemokine receptors and adhesion proteins, whereas T_CM_ shares certain cell plasma membrane markers with naive T cells (28). The proportion of T cells with a CM phenotype, compared with those of the EM phenotype, was higher in each group (**Figures 3A–D**; **Supplementary Figure 4**). Within the total T cell population, the frequency of CM was higher than that of EM (**Supplementary Table 1**). Compared with control T cells, the proportion of PB CAR-T cells with an EM phenotype (**Figure 3A**), or CM phenotype, was lower in the total cell population and CD4^+^ subsets (**Figures 3A, B**). No significant difference was observed in memory phenotypes among the CD8^+^ and DN subsets (**Figures 3C, D**). Collectively, these data suggest that fewer PB CAR-T cells exhibit a pronounced memory phenotype, which may be due to the susceptibility of memory T cells to electroporation in the manufacturing process (29).

### 3.4 Transcriptomic comparison of Lenti and PB CAR-T cells

**Figure 4:**
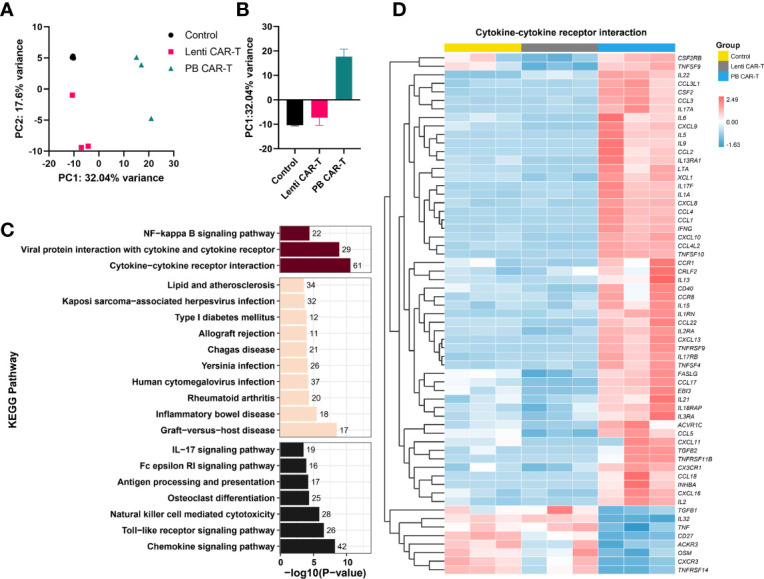
Transcriptome analysis. CAR-T cells were sorted on day 3 post-transfection and then rested for 24 hours. The total RNA of sorted CAR-T cells was analyzed using RNA-seq. **(A, B)** PCA analysis of control T cells, PB CAR-T cells, and Lenti CAR-T cells (*n* =3). **(C)** Kyoto Encyclopedia of Genes and Genomes (KEGG) pathway analysis (PB CAR-T cells *vs.* Lenti CAR-T cells). The purplish red bar represents environmental information processing, the beige bar represents human diseases, and the black bar represents organismal systems. **(D)** Hierarchical cluster analysis for the “cytokine-cytokine receptor interaction” pathway (KEGG enrichment).

To identify transcriptome-wide alterations induced by the different manufacturing methods, RNA-sequencing (RNA-seq) was conducted on samples obtained from three healthy donors. PCA analysis revealed a marked transcriptomic difference between Lenti and PB CAR-T cells (**Figures 4A, B**), suggesting that Lenti and PB CAR-T cells may be two distinct cell populations despite their similar flow cytometry profiles. To gain a more comprehensive understanding of the functions of differentially expressed genes in PB CAR-T cells compared to Lenti CAR-T cells, we conducted KEGG pathway annotations and enrichment analysis. Our results revealed that 67 pathways were significantly overrepresented (adjusted *P* < 0.05), including “cytokine-cytokine receptor interaction”, “chemokine signaling pathway,” and “viral protein interaction with cytokine and cytokine receptor” (**Figure 4C**). T cells execute their functions primarily by secreting various cytokines and chemokines (23). The hierarchical clustering (**Figure 4D**) and GSEA analysis (**Supplementary Figure 5**) of “cytokine-cytokine receptor interaction” pathway revealed the upregulated cytokine synthesis in PB CAR-T. To further validate the transcriptomic difference between the two CAR-Ts, we employed RT-qPCR to quantify the transcription of upregulated genes in PB CAR-T. The RT-qPCR assays confirmed that the mRNA levels of *gm-csf*, *cxcl13*, *ifn-γ*, *il9*, *serpine1*, *il17f*, *il3*, and *ccl22* were higher in the PB CAR-T groups than the Control T groups. Furthermore, in comparison to Lenti CAR-T cells, the majority of these genes exhibited elevated mRNA levels in PB CAR-T cells (as demonstrated in **Supplementary Figure 6**). These findings suggest that PB CAR-T cells may possess a proinflammatory phenotype.

### 3.5 PB CAR-T cells create an intensive cytokine microenvironment *in vitro*

**Figure 5:**
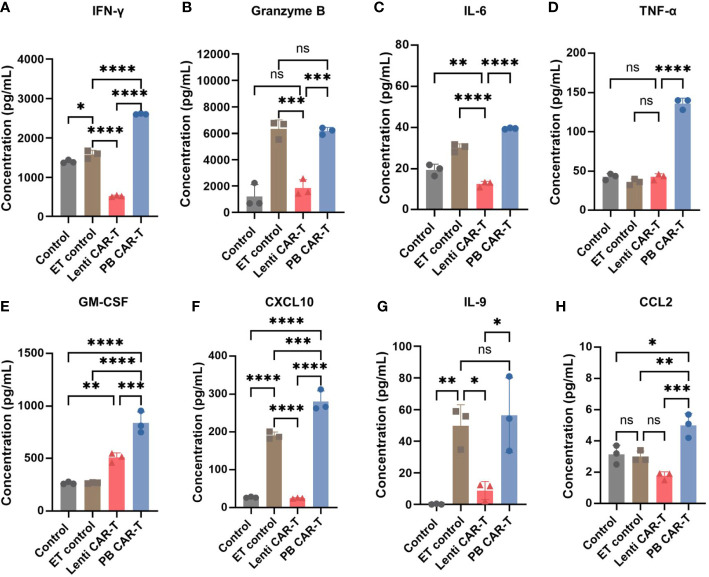
Cytokine expression profiles in control T cells, empty transfection (ET) control T cells, Lenti CAR-T cells, and PB CAR-T cells. CAR-T cells were sorted on day 3 post-transfection and rested for 24 hours. ET control T cells were transfected with an empty transposon vector using electroporation, with a total amount of 2.1 μg. Concentrations of IFN-γ **(A)**, granzyme B **(B)**, IL-6 **(C)**, TNF-α **(D)**, GM-CSF **(E)**, CXCL10 **(F)**, IL-9 **(G)**, and CCL2 **(H)** in the medium determined by ELISA (*n* = 3). One-way ANOVA with Tukey’s multiple comparison test. *P < 0.05, **P < 0.01, ***P < 0.001, ****P < 0.0001, ns indicates not significant (*P >*0.05).

To validate the differences detected by RNA-seq, we performed a quantitative cytokine analysis using ELISA. Numerous cytokines were released into the media by PB CAR-T cells, including IFN-γ, granzyme B, interleukin (IL)-6, tumor necrosis factor (TNF)-α, Granulocyte-macrophage colony-stimulating factor (GM-CSF), CXCL10, IL-9, and CCL2 (**Figures 5A–H**). Of these, IL-6, IL-9, IFN-γ, TNF-α, and GM-CSF are critical soluble mediators of cytokine storms (30). These data suggested that the electroporation of PB transposon vectors induced the release of numerous high-concentration cytokines and a cytokine storm-like microenvironment *in vitro*, which differed markedly from the cytokine profile associated with CAR-T cells produced via lentiviral transfection. To further investigate the effects of the electroporation process, we established a control group in which T cells were electroporated with an empty vector. Higher concentrations of granzyme B (**Figure 5B**), CXCL10 (**Figure 5F**), and IL-9 (**Figure 5G**) were observed in this control group, indicating that the electroporation process may have partially contributed to the proinflammatory phenotype of PB CAR-T cells.

### 3.6 *In vitro* assessment of anti-tumor efficacy

**Figure 6:**
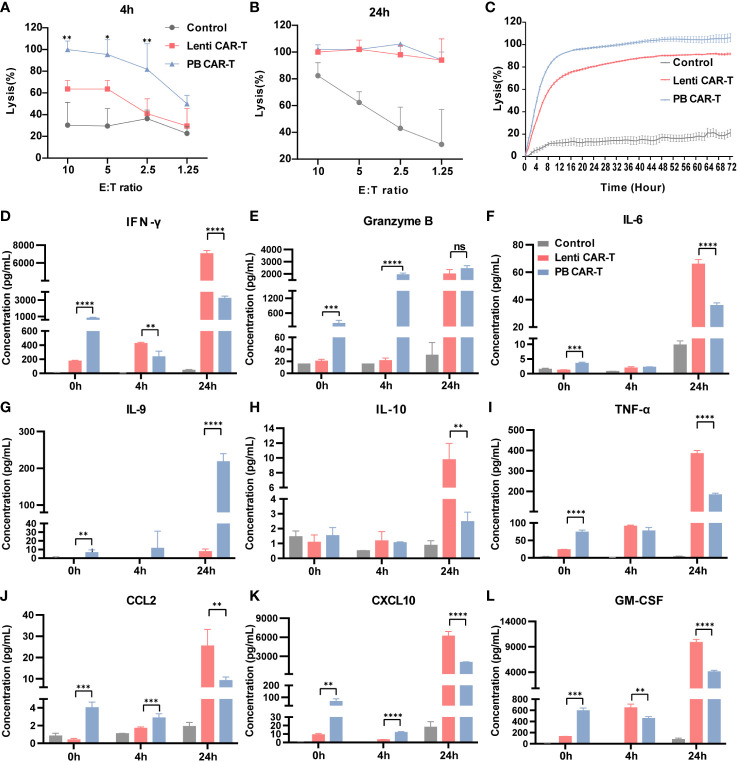
*In vitro* anti-tumor efficacy of PB and Lenti CAR-T cells. CAR-T cells at day 7 post-transfection were tested for anti-tumor efficacy. K562-CD19-luc cells expressing luciferase were used as target cells. **(A, B)** Anti-tumor efficacy determined at 4 hours **(A)** and 24 hours **(B)** post-co-culture. **(C)** Continuous monitoring of the anti-tumor efficacy determined based on the RTCA platform for 72 hours (E:T = 2.5:1). **(D–L)** Detection of human cytokines and chemokines. Concentrations of IFN-γ **(D)**, granzyme B **(E)**, IL-6 **(F)**, IL-9 **(G)**, IL-10 **(H)**, TNF-α **(I)**, CCL2 **(J)**, CXCL10 **(K)** and GM-CSF **(L)** in the medium before co-culture and at 4 and 24 hours post-coculture determined by Ella automated immunoassay or ELISA (*n* = 3). Significance was determined using an unpaired *t’*-test for **(A**, **B)** One-way ANOVA with Tukey’s multiple comparisons test for **(D–L)**. *P < 0.05, **P < 0.01, ***P < 0.001, ****P < 0.0001, ns, not significant (*P >*0.05).

The CD19-expressing luciferase-tagged K562 human leukemia cell line was used as target cells to assess CAR-T and T cell anti-tumor efficacy. K562 cell cytotoxicity in the presence of CAR-T and T cells was evaluated using a luciferin-based assay (31). The results showed that at 4 hours post-co-culture, PB CAR-T cells (effector) induced significantly higher K562 cell (target) cytotoxicity than Lenti CAR-T cells at all effector-to-target ratios (E/T ratios) tested, except for an E/T ratio of 1.25 (**Figure 6A**). However, at 24 hours post-co-culture, PB CAR-T cells induced similar levels of K562 cell cytotoxicity as Lenti CAR-T cells (**Figure 6B**). Subsequently, we utilized the RTCA (xCELLigence Real-Time Cell Analyzer) method to continuously monitor the cytotoxicity of CAR-T cells in real-time. The RTCA assay revealed that PB CAR-T cells exhibited a more rapid cytotoxic effect on K562 compared to Lenti CAR-T cells at an E/T ratio of 2.5 (**Figure 6C**), which was consistent with the results obtained from the luciferin-based assay.

To better understand the difference in the overall cytotoxicity caused by PB and Lenti CAR-T cells, the abundance of numerous cytokines was assessed before and after the cytotoxicity assay (**Figures 6D–L**). Prior to co-culture, PB CAR-T cells exhibited a proinflammatory phenotype with increased secretion of cytokines, as suggested by the results of RNA-seq. However, at 4 hours post co-culture, Lenti CAR-T cells higher levels of IFN-γ (**Figure 6D**) and GM-CSF (**Figure 6L**). Furthermore, at 24 hours post co-culture, Lenti CAR-T cells secreted higher amounts of all tested cytokines except for granzyme B (**Figure 6E**) and IL-9 (**Figure 6G**), which was consistent with previous work demonstrating the cytokine release syndrome elicited by Lenti CAR-T cells *in vivo* (13). The rapid and effective cytotoxicity of PB CAR-T cells may be attributed to their initial proinflammatory phenotype and subsequent substantial secretion of IL-9 upon contact with target cells. IL-9 is reportedly important in the anti-tumor immune response (32).

### 3.7 *In vivo* assessment of anti-tumor efficacy

**Figure 7:**
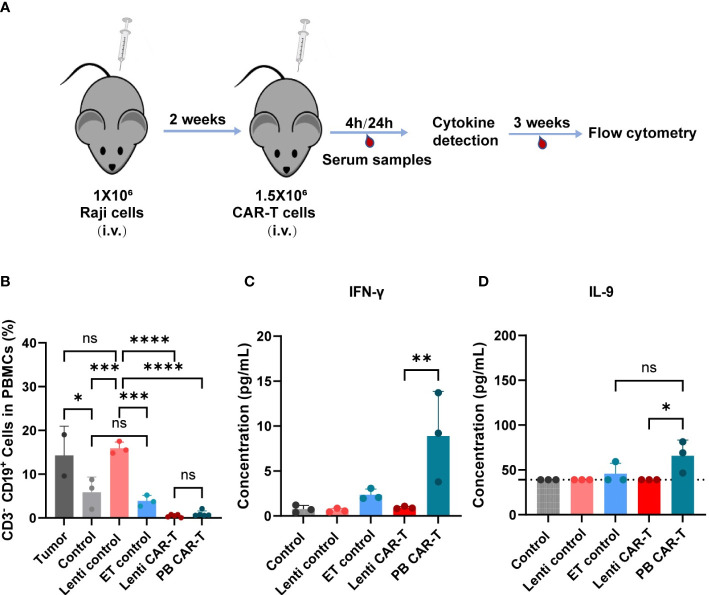
*In vivo* anti-tumor efficacy and cytokine production of PB and Lenti CAR-T. **(A)** Schematic of the mouse model. The Raji cells are injected into the tail vein of mice and are allowed to grow for two weeks after injection. Then 1.5 million CAR-T cells were transferred into the mice *via* tail vein injection on Day 14. After 4 hours and 24 hours, serum samples were collected for cytokine detection *via* facial vein. For three weeks, blood was collected for flow cytometry analysis. **(B)** Percentages of human CD3^-^ CD19^+^ cells in mouse PBMCs collected on day 21 after CAR-T infusion determined via Flow cytometry. (Tumor group n=3, *one died; Lenti control and ET control groups n=3; control group n=5, *two died; Lenti CAR-T group n=5; PB CAR-T group n=5). The concentration of human IFN-γ **(C)** and IL-9 **(D)** in serum determined at 4 hours after PB and Lenti CAR-T infusion by ELISA or Ella automated immunoassay. The limit of detection of IL-9 ELISA kits was 39.0 pg/mL and presented as dotted lines. One-way ANOVA with Tukey’s multiple comparison test. *P < 0.05, **P < 0.01, ***P < 0.001, ****P < 0.0001, ns indicates not significant (*P >*0.05). All data were presented as mean ± standard deviation.

To evaluate the effects of two CAR-Ts on the anti-tumor response, we conducted evaluation of their *in vivo* efficacy (**Figure 7A**). Specifically, we collected blood PBMC from euthanized NSG (NOD-*Prkdc^scid^Il2rg^em1^*/Smoc) mice, staining with anti-hCD19 antibody, and analyzed them using flow cytometry. Both PB and Lenti CAR-T groups exhibited a significant reduction in the percentage of CD19^+^ CD3^-^ cells, with no significant difference observed between the two groups (refer to **Figure 7B**). Furthermore, we quantified the concentrations of cytokines secreted by human T/CAR-T cells in the serum of mice. The results demonstrated that the concentrations of IFN-γ and IL-9 were augmented in PB CAR-T cells relative to Lenti CAR-T cells at 4 hours post-infusion (**Figures 7C, D**), which subsequently declined to levels below the limit of detection at 24 hours (data not shown). Conversely, all other cytokines, including IL-6, IL-10, IL-12, GM-CSF, TNF-α, CXCL10 and CCL2, were undetectable at both 4 and 24 hours. Overall, these data suggested that PB CAR-T and Lenti CAR-T exhibit comparable *in vivo* anti-tumor efficacy.

## 4 Discussion

Genetically modified T-cell therapy is a promising and innovative treatment for deleterious leukemia (5). Previous studies have proposed several methods of gene modification to express CARs in T cells, including the prevailing lentivirus/retrovirus and PiggyBac transposon systems (13). In this study, we conducted a rigorous comparative analysis to assess the disparities between Lenti and PB CAR-T cells. Our findings indicated that these two types of CAR-T cells were distinct in terms of their transcriptome and cytokine secretion. Specifically, the PB CAR-T cells exhibited more rapid *in vitro* anti-tumor activity compared to Lenti CAR-T cells, while demonstrating similar tumor eradication ability *in vivo* as Lenti CAR-T cells.

For our analyses, the positivity rates of CAR-T cells manufactured by lentivirus and electroporation were adjusted to a similar level (approximately 30%) to reduce potential confounding variables. Interestingly, at similar CAR positivity rates, PB CAR-T cells exhibited significantly brighter green fluorescence in certain cells than Lenti CAR-T cells, indicating higher CAR expression, which was confirmed by the Protein L staining assay. We postulate that the distinct integration sites (21) may have contributed to the divergent CAR expression profiles. Notably, during lentiviral transfection, several viral components were co-integrated into target cells with the CAR ORF (33–35), which may have hindered the transcriptional efficiency of the target gene.

The number of CM T cells is related to the effectiveness of CAR-T cell therapy (36). CAR-T cells manufactured using the transposon system were previously reported to exhibit a CM phenotype elicited by 4-1BB co-stimulation signaling (37). Moreover, CD4^+^ CAR-T cells were recently reported to be the dominant cells for persistent remission in leukemia patients treated with CAR-T cell therapy (38). Our data demonstrated that the proportion of CD4^+^ CM T cell subsets remained highest among subsets after CAR transfection via both methods. For CD4^+^ CM subsets, PB CAR-T was lower than those in control CAR-T. Although no significant difference was observed between Lenti and PB CAR-T, the mean values of CD4^+^ CM cells in Lenti CAR-T were higher than those in PB CAR-T. In addition, the frequency of CD8^+^ memory phenotypes did not differ. Furthermore, in total subsets, the proportion of memory phenotypes of PB CAR-T cells was the lowest among the three groups, which may imply electroporation-associated toxicity against memory phenotypes (29). Taken together, Lenti CAR-T may be more durable than PB CAR-T after infusion due to their induction of T cell memory phenotypes.

An important finding of our study is that PB CAR-T cells exhibited higher basal cytokine levels than Lenti CAR-T cells prior to co-culture with target cells. This result was further supported by our RNA-seq data, which detected elevated expression of cytokines and chemokines in PB CAR-T cells. Moreover, upon antigenic activation *in vitro*, PB CAR-T cells released lower levels of IFN-γ, IL-6, IL-10, TNF-α, CCL2, CXCL10, and GM-CSF than Lenti CAR-T cells, but demonstrated a robust release of IL-9, indicative of a distinct anti-tumor response pathway. In the *in vivo* experiments, the levels of cytokines were not as significant as those observed *in vitro*, possibly due to the lower secretion levels of cytokines that were not effectively detectable.

IL-9, a T-cell growth factor, is a member of the γ-chain-receptor cytokine family and is secreted by Th2 (T helper 2), Th9 (T helper 9), Th17 (T helper 17), and NKT (natural killer T) cells (39). IL-9 has been demonstrated to increase the longevity of Tc9 cells (40). Importantly, the IL-9 signaling pathway has been found to be particularly effective in enhancing the anti-tumor response of CAR-T cells (41). Our data revealed that the PB transposon system upregulates IL-9 expression in T cells. Although IL-9 expression was reduced by day 7 post-transfection, it was upregulated again in PB CAR-T cells upon encountering tumor cells. The mechanisms underlying IL-9 upregulation in PB CAR-T cells remain to be elucidated.

In comparison to the safety of lentiviral/retroviral systems observed in a cohort of 308 patients (16), a recent clinical trial on PB CAR-T-cell lymphoma reported a concerning oncogenic effect of transposon gene integration system (42). In this study, we did not observe differentially expressed genes in PB CAR-T cells (versus Lenti CAR-T) that were significantly enriched in tumor-associated signaling pathways. However, a small fraction of PB CAR-T cells exhibited a strong green fluorescent signal under similar proportions of CAR positivity (**Figure 1E**), indicating that the number of inserted CAR copies may be high in a few PB CAR-T cells, which may increase the risk of oncogenic insertion mutagenesis. The oncogenic potential of PB CAR-T cells requires further investigation via single-cell RNA-seq or insertion mutagenesis analysis.

IL-6 is secreted by various immune and stromal cells and exerts multiple functions (43). IL-6 is considered a hub cytokine in CRS triggered by CAR-T cell therapy (30). In fact, the prognosis of CRS was improved by tocilizumab, an IL-6 receptor (IL-6R) monoclonal antibody that blocks IL-6 binding to IL-6R (44). In our study, secretion of IL-6 by PB and Lenti CAR-T cells was unchanged at 4 hours after encountering tumor cells, however, Lenti CAR-T cells released much higher levels of IL-6 into the media than PB CAR-T cells at 24 hours. These data indicate that PB CAR-T may cause less CRS in clinical applications; this conclusion is further supported by a recent report on CAR-T cells manufactured via electroporation (45).

It is important to note that the original PBMCs used to produce both CAR-T cell groups were from several donors; therefore, it will be important to further validate our findings in an expanded group of donors. Despite this limitation, these data revealed a large disparity arising from the two main manufacturing methods used to produce CAR-T cells. These findings shed new light on the effect of different production methods on the phenotypes of seemingly similar cell types and will inform the design of future cell-based therapies.

## Data availability statement

The datasets presented in this study can be found in online repositories. The names of the repository/repositories and accession number(s) can be found below: NCBI Gene Expression Omnibus under the accession number GEO: GSE212072.

## Ethics statement

The donors’ privacy was protected and the study protocol complied with The Helsinki Declaration. All donors provided written informed consent, and this study was approved by Ethics Committees (approval number: AF/SC-08/02.258).

## Author contributions

Conceived and designed the experiments: WC, XZ, JZ, and AN; analyzed the data: AN, JZ, and XH; contributed reagents/materials: ZZ, LS, JW, XL and WZ; wrote and revised the paper: AN, JZ, WC, and XZ. All authors contributed to the article and approved the submitted version.
