# Supplementary material for: Differences in the phenotypes and transcriptomic signatures of chimeric antigen receptor T lymphocytes manufactured via electroporation or lentiviral transfection
Source: Front Immunol. 2023 May 9;14:1068625. doi: 10.3389/fimmu.2023.1068625 (PMC10203401; doi:10.3389/fimmu.2023.1068625)
Supplement: Supplementary file 1 [file DataSheet_1.docx]

Supplementary Material

Differences in the phenotypes and transcriptomic signatures of chimeric antigen receptor T lymphocytes manufactured via electroporation or lentiviral transfection

Anna Niu^†^, Jintao Zou^†^, Xuan Hu, Zhang Zhang, Lingyu Su, Jing Wang, Xing Lu, Wei Zhang, Wei Chen^*^, Xiaopeng Zhang^*^

^†^ These authors contributed equally.

* Correspondence: Wei Chen: cw0226@foxmail.com; Xiaopeng Zhang: zhangxp@bmi.ac.cn

# Supplementary Figures and Tables

## Supplementary Figures


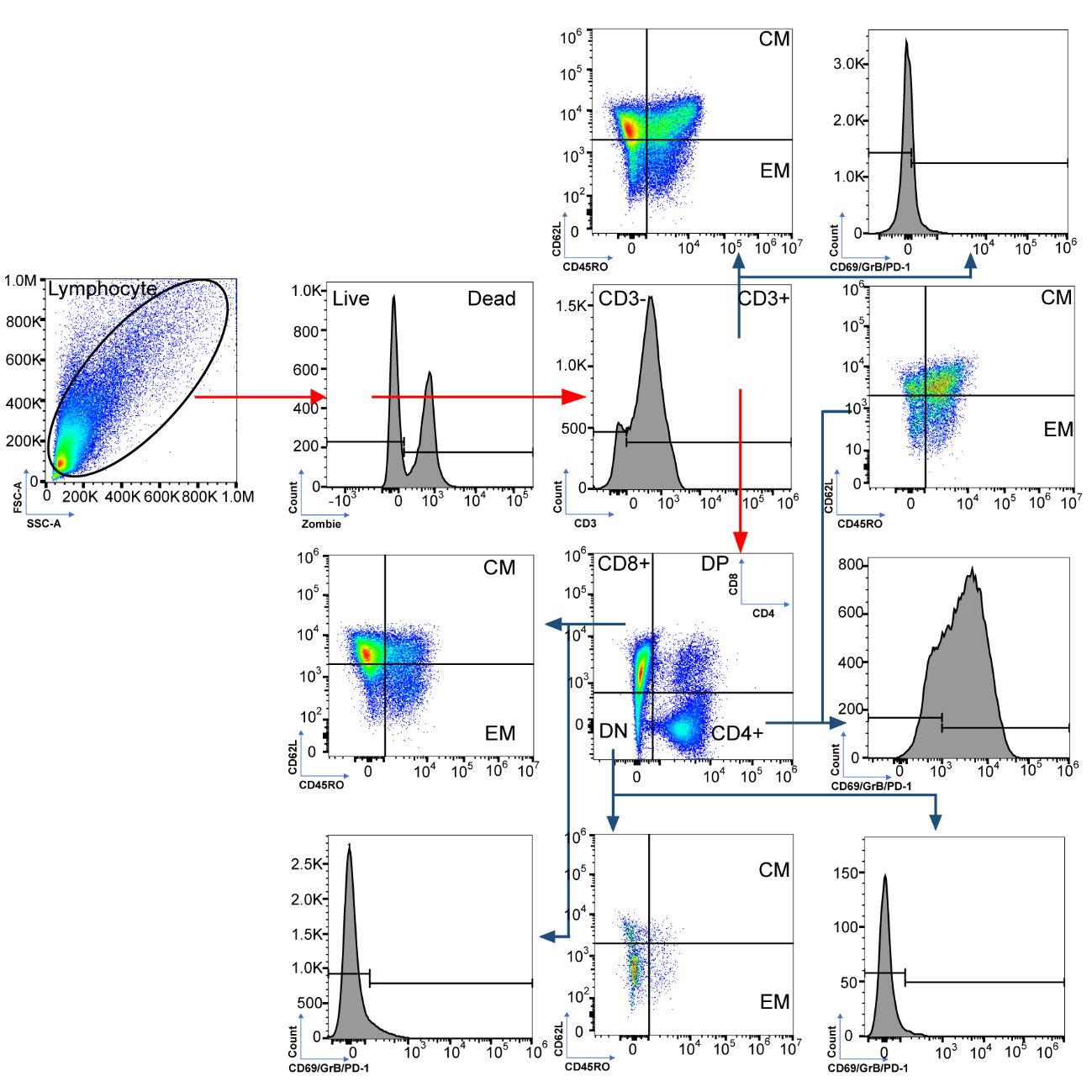


**Supplementary Figure 1.** Representative flow cytometry gating strategy for T cell phenotyping. Total lymphocytes were gated based on forward scatter/side scatter (FSC/SSC) plot, followed by gating against Zombie dye to exclude dead cells. T cell subsets were identified by gating CD3^+^ cells, and then distinguished based on CD4 and CD8 expression. These populations were further analyzed for CD69 expression (activation marker), granzyme B expression (cytotoxic marker), and PD-1 (exhaustion marker). Memory T cells were identified by gating CD45RO^+^ cells and further distinguished based on CD62L expression.

**
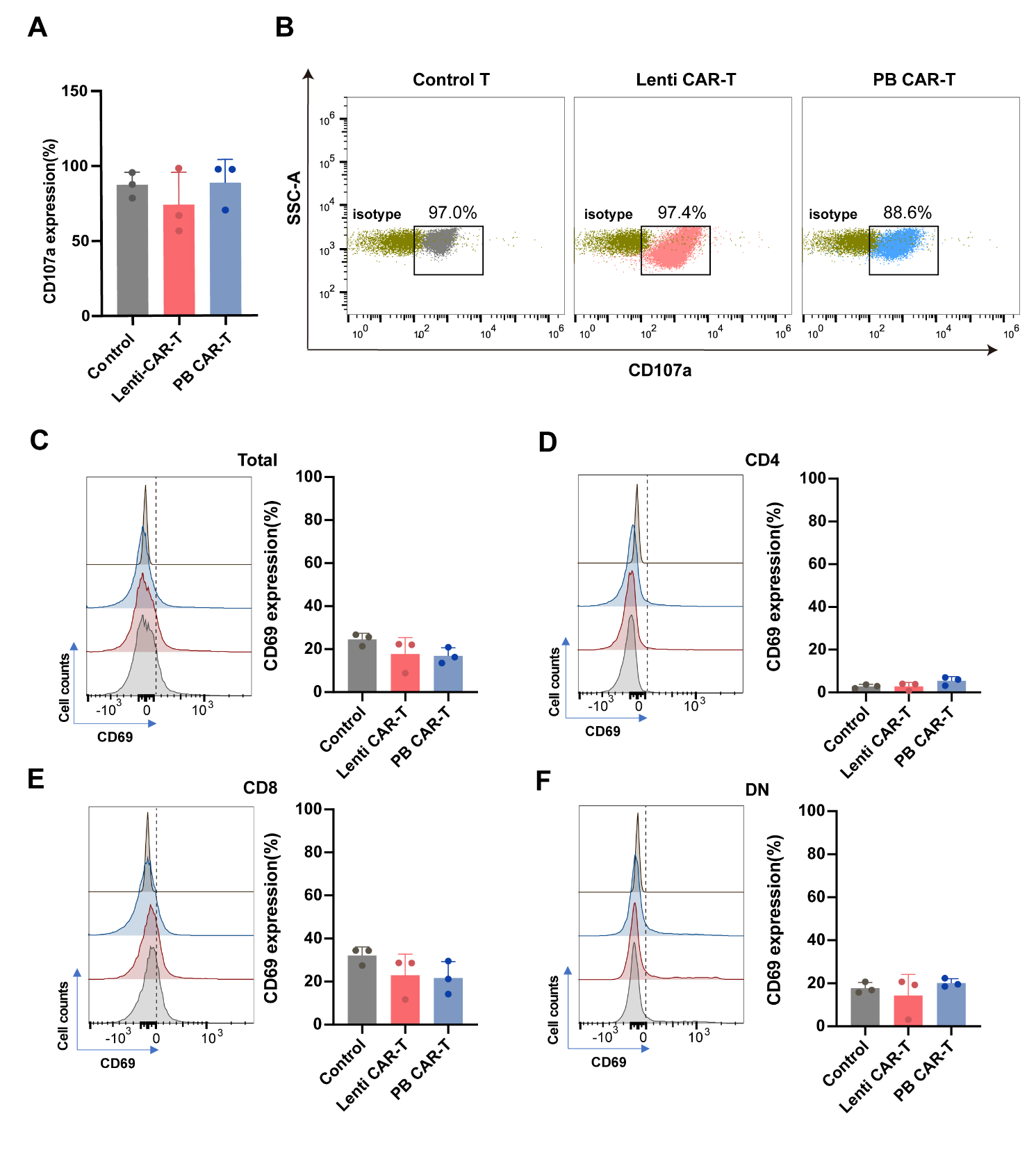
**

**Supplementary Figure 2.** CD107a and CD69 expression of total T cells at day 3 post-transfection. Representative histograms and statistical analysis of CD107a expression in total T cells **(A)** and scatter dots plot of CD107a **(B)** in control, Lenti, and PB CAR-T cells. Representative histogram images and statistical analysis of CD69 expressions in total T cells **(C)** and CD4^+^ **(D)**, CD8^+^ **(E)**, and double negative (DN) **(F)** T cell subsets (*n* = 3). Isotype control were presented in the upper first lane. One-way ANOVA with Tukey’s multiple comparisons test.


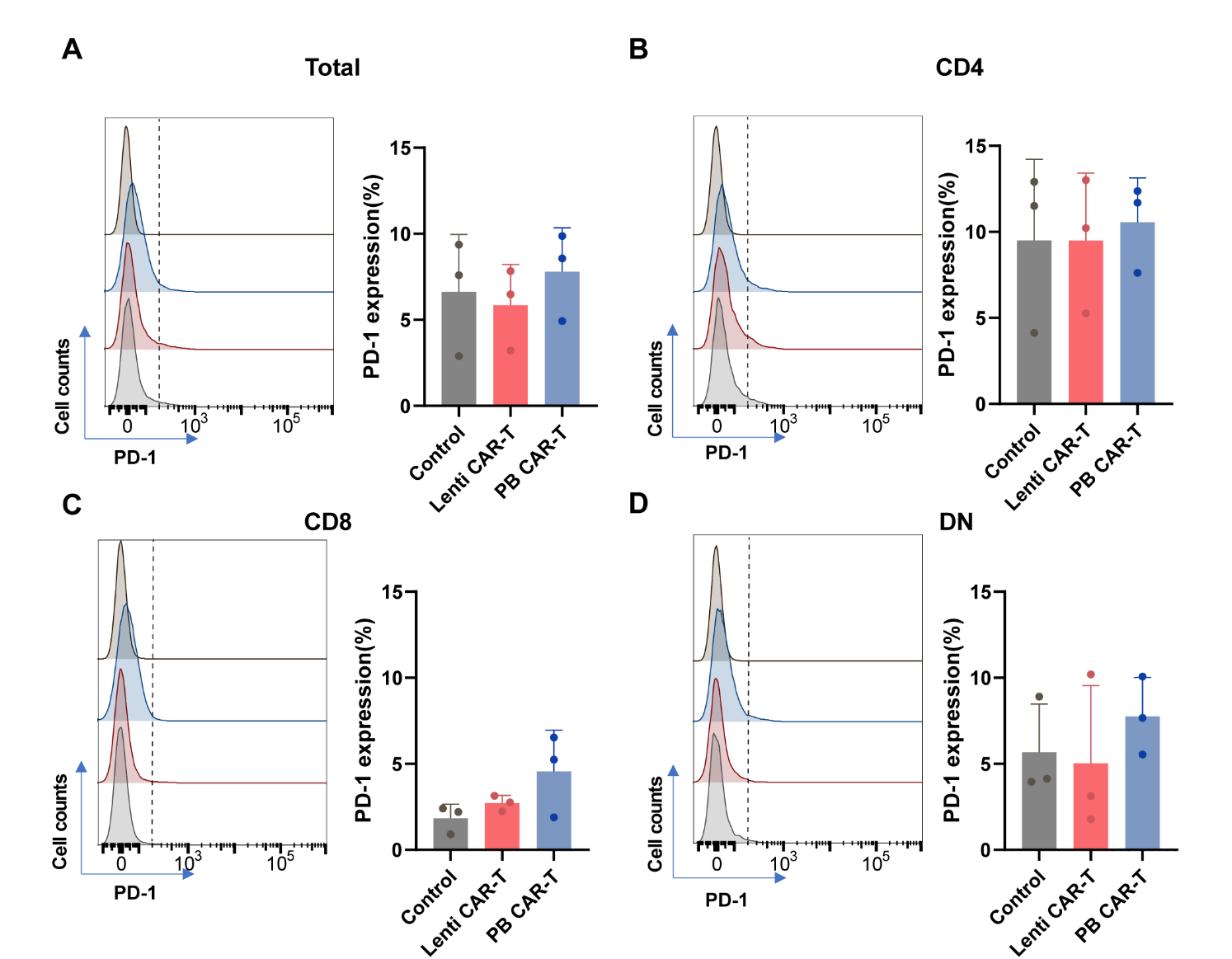


**Supplementary Figure 3**. Exhaustion of total T cells and T cell subsets at day 3 post transfection. Representative histograms and statistical analysis of PD-1 expression in total T cells **(A)**, CD4^+^ T cells **(B)**, CD8^+^ T cells **(C)**, and DN T cells **(D)**. Isotype control were presented in the upper first lane. Gate for PD-1 was established with fluorescence minus one control. The representative histogram plot was provided by one of three donors and bar graphs are presented as the mean for three donors with standard deviation (*n* = 3).


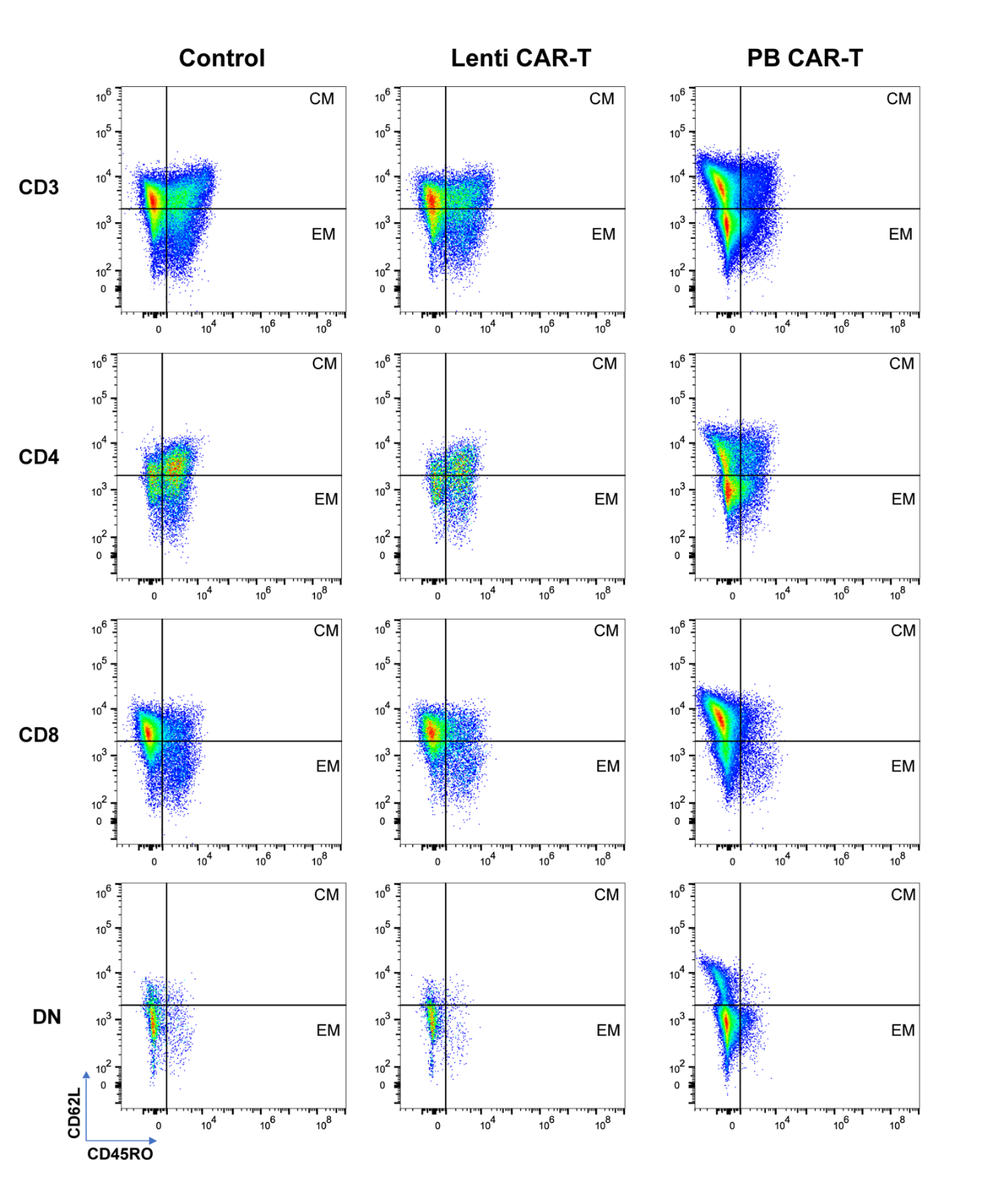


**Supplementary Figure 4.** Representative images of CD45RO and CD62L expression in total T cells, CD4^+^ T cells, CD8^+^ T cells, and DN T cells at day 3 post transfection. An effector memory (EM) phenotype corresponds to CD45RO^+^CD62L^-^ and a central memory (CM) phenotype corresponds to CD45RO^+^CD62L^+^.


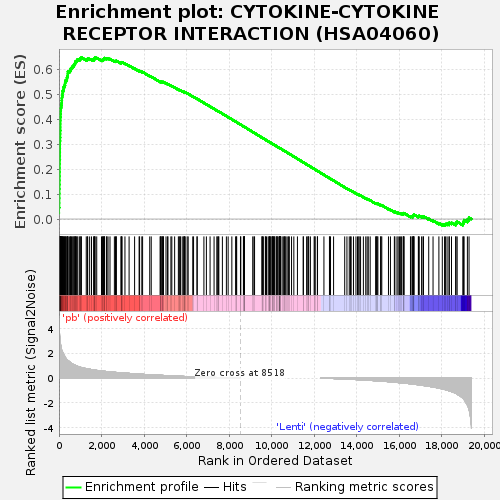


**Supplementary Figure 5.** Gene set enrichment analysis (GSEA) analysis of "cytokine-cytokine receptor interaction" pathway in PB CAR-T cells *vs* Lenti CAR-T cells.


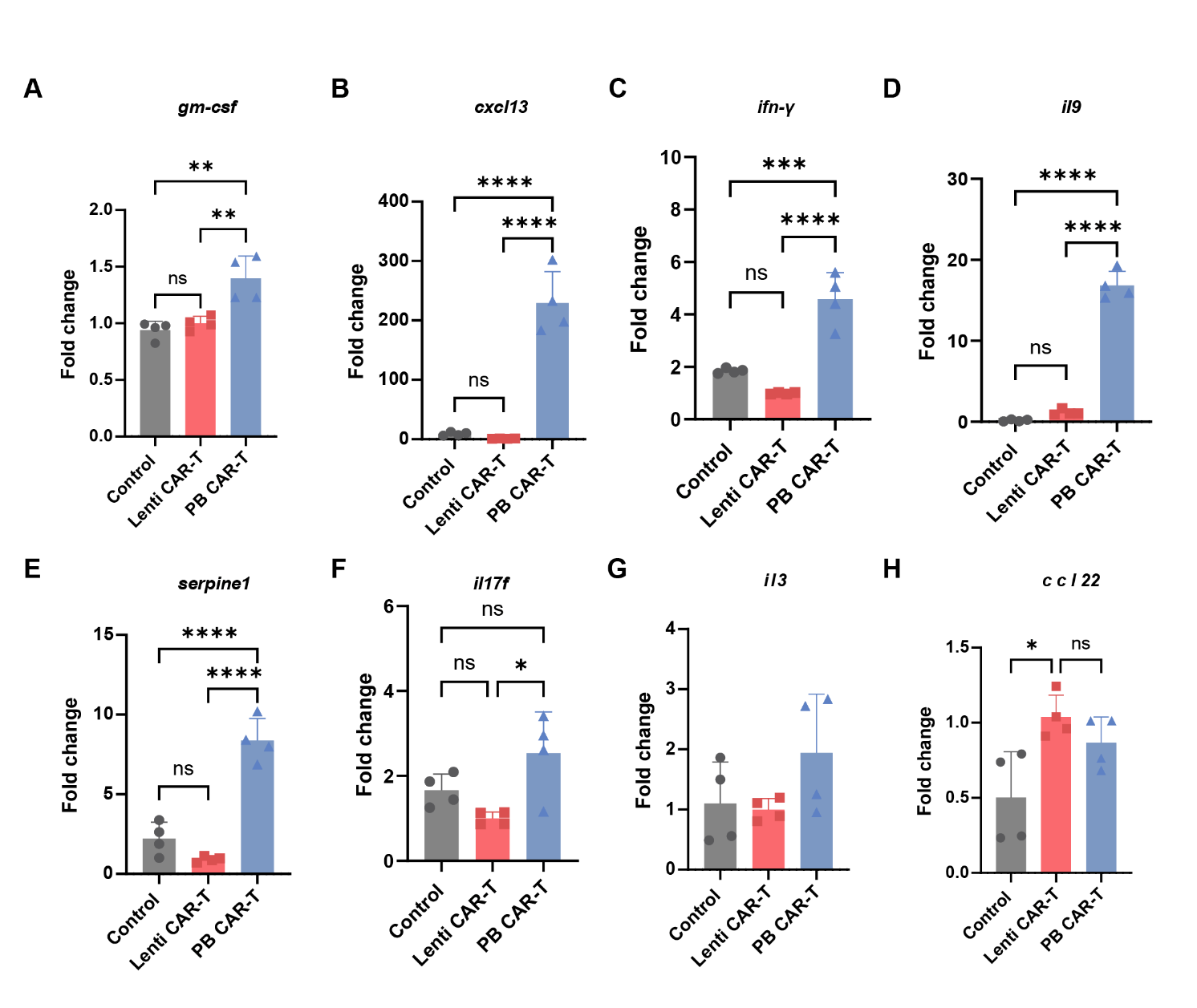


**Supplementary Figure 6.** Validation of transcriptional alterations of genes determined via RT-qPCR. (A) *gm-csf*. (B) *cxcl13*. (C) *ifn-γ*. (D) *il9*. (E) *serpine1*. (F)*il17f.* (G)*il3*. (H)*ccl22*. The normalized fold expression of each gene was obtained from four independent experiments and represented in each bar as the mean ±standard deviation. One-way ANOVA with Tukey’s multiple comparisons test. **P <* 0.05, ***P <* 0.01, ***P <* 0.01, ****P <* 0.001, *****P <* 0.0001, ns indicates not significant (*P* >0.05).

## Supplementary Tables

**Supplementary Table 1** Effector and central memory phenotypes of total T cells and T cell subsets

| **Group** | **CD3** | | **CD4** | | **CD8** | | **DN** | |
| --- | --- | --- | --- | --- | --- | --- | --- | --- |
|  | **EM** | **CM** | **EM** | **CM** | **EM** | **CM** | **EM** | **CM** |
| Control | 15.67 | 23.17 | 28.03 | 40.47 | 11.30 | 9.29 | 9.91 | 3.24 |
|  | 14.03 | 30.63 | 24.77 | 46.13 | 10.67 | 11.67 | 12.73 | 4.92 |
|  | 7.72 | 20.43 | 10.93 | 27.23 | 7.02 | 13.90 | 6.33 | 5.91 |
| Lenti CAR-T | 17.73 | 21.93 | 31.13 | 34.83 | 13.17 | 12.13 | 7.00 | 2.36 |
|  | 4.44 | 11.70 | 4.81 | 22.03 | 4.17 | 7.47 | 5.61 | 9.17 |
|  | 4.79 | 9.49 | 7.77 | 17.20 | 3.69 | 4.75 | 3.80 | 1.73 |
| PB CAR-T | 5.56 | 6.38 | 12.23 | 11.30 | 2.41 | 3.34 | 3.98 | 0.53 |
|  | 4.53 | 15.93 | 5.82 | 26.30 | 3.93 | 7.48 | 4.71 | 3.31 |
|  | 4.37 | 5.96 | 5.30 | 9.43 | 3.85 | 3.12 | 3.20 | 0.58 |
